# *SHANK3* haploinsufficiency: a “common” but underdiagnosed highly penetrant monogenic cause of autism spectrum disorders

**DOI:** 10.1186/2040-2392-4-17

**Published:** 2013-06-11

**Authors:** Catalina Betancur, Joseph D Buxbaum

**Affiliations:** 1 INSERM U952, Paris, France; 2 CNRS UMR 7224, Paris, France; 3 Université Pierre et Marie Curie, Paris, France; 4 Seaver Autism Center for Research and Treatment, Departments of Psychiatry, Neuroscience, and Genetics and Genomic Sciences, Friedman Brain Institute, and Mindich Child Health and Development Institute, Icahn School of Medicine at Mount Sinai, New York, NY 10029, USA

## Abstract

Autism spectrum disorders (ASD) are etiologically heterogeneous, with hundreds of rare, highly penetrant mutations and genomic imbalances involved, each contributing to a very small fraction of cases. In this issue of *Molecular Autism*, Soorya and colleagues evaluated 32 patients with Phelan-McDermid syndrome, caused by either deletion of 22q13.33 or *SHANK3* mutations, using gold-standard diagnostic assessments and showed that 84% met criteria for ASD, including 75% meeting criteria for autism. This study and prior studies demonstrate that this syndrome appears to be one of the more penetrant causes of ASD. In this companion review, we show that in samples ascertained for ASD, *SHANK3* haploinsufficiency is one of the more prevalent monogenic causes of ASD, explaining at least 0.5% of cases. We note that *SHANK3* haploinsufficiency remains underdiagnosed in ASD and developmental delay, although with the increasingly widespread use of chromosomal microarray analysis and targeted sequencing of *SHANK3*, the number of cases is bound to rise.

## 

Autism spectrum disorders (ASD) are highly genetic disorders, and current estimates indicate that there could be over 1,000 genes that contribute to ASD risk [1]. Very few genes are therefore likely to contribute to more than 1% of ASD, and mutations of *FMR1* (the gene disrupted in Fragile X syndrome) and *MECP2* (the gene disrupted in Rett syndrome), considered among the most common causes of ASD, explain 2% and 0.5% of ASD, respectively. Here we show that loss of a functional copy of *SHANK3* is among the more prevalent rare causes of ASD.

*SHANK3* codes for a scaffolding protein that lies at the core of the postsynaptic density in glutamatergic synapses. 22q13.3 deletions and mutations that lead to a loss of a functional copy of *SHANK3* cause Phelan-McDermid syndrome, characterized by moderate to profound intellectual disability, severely delayed or absent speech, hypotonia, and ASD or ASD traits [2,3]. Dysmorphic features are usually mild and include dysplastic nails, large or prominent ears, long eyelashes, wide nasal bridge, bulbous nose and sacral dimple. Decreased perspiration, mouthing or chewing non-food items, and decreased perception of pain are frequently noted. Other features include seizures, brain, renal and cardiac malformations, motor deficits, gastroesophageal reflux, lymphedema, and immune defects. Because of its nonspecific clinical presentation, the diagnosis requires molecular genetic testing to identify *SHANK3* deletions (the preferred method being chromosome microarray analysis) or mutations.

In this issue, Soorya and colleagues evaluated ASD in a sample of 32 patients with *SHANK3* haploinsufficiency using standard diagnostic tests — the Autism Diagnostic Interview-Revised and the Autism Diagnostic Observation Schedule — and showed that 84% (27/32) met criteria for ASD, including 75% (24/32) meeting criteria for autism. These findings indicate that Phelan-McDermid syndrome is one of the more highly penetrant causes of autism [4].

We can get a reasonably accurate estimate of the frequency of *SHANK3* deletions and mutations in ASD through the review of recent studies in ASD that made use of either chromosome microarray or targeted resequencing of *SHANK3*. A survey of all relevant studies, including negative studies, indicates that at least 0.5% of subjects with ASD have haploinsufficiency at the *SHANK3* locus. Table 1 shows 14 genome-wide microrray studies in ASD that would reliably detect larger dosage imbalance at *SHANK3*. These studies included 7,887 affected individuals, and collectively identified 13 deletions (0.16%). This frequency is likely underestimated because, in many of these studies, efforts were made at the recruiting sites to exclude cases with severe intellectual disability or syndromic autism (that is, those with dysmorphic features or other congenital anomalies). In addition, many of the patient samples had been prescreened for cytogenetic abnormalities and microdeletion/microduplication syndromes. Furthermore, although we tried to exclude studies that had clearly overlapping samples, there are probable sample overlaps among the remaining studies (overlapping ASD cases without a deletion would lead to apparently decreased rates of the deletion). Moreover, because Phelan-McDermid syndrome is a mostly sporadic disorder (the deletion is *de novo* in 80% of cases, while in 20% it results from familial balanced translocations or other chromosome rearrangements), screening ASD samples with an overrepresentation of multiplex families will necessarily result in a lower yield. Finally, it should be noted that most of the microarray analyses reviewed here would have missed small deletions involving only *SHANK3.*

**Table 1:** **22q13.3 deletions involving *SHANK3 *identified through microarray analyses in autism spectrum disorder samples**

**Study**	**Subjects**	**22q13.3 deletions**
Sebat *et al.*[5]	165	1 *de novo*
Moessner *et al*. [6]	400	2 *de novo*^a^
Weiss *et al*. [7]	299 ^b^	0
van der Zwaag *et al.*[8]	105	0
Guilmatre *et al.*[9]	260	2 *de novo*
Qiao *et al.*[10]	100	0
Schaefer *et al.*[11]	68	0
Pinto *et al.*[12] + Autism Genome Project (manuscript in preparation)	2,446	3 *de novo*^c^
Shen *et al.*[13]	848	0
Rosenfeld *et al.*[14]	1,461	4 (2 *de novo*, 2 unknown)
Bremer *et al.*[15]	223	1 *de novo*
Sanders *et al.*[16]	1,124	0
Wisniowiecka-Kowalnik *et al.*[17]	145	0
Girirajan *et al.*[18]	243	0
**Total**	**7,887**	**13 (0.16%)**

^a^ Family 3524, with two affected siblings with an apparent de novo *SHANK3* deletion, was part of another cohort and was thus not included here. In addition, this family’s deletion was previously reported in Sebat *et al*. [5].

^b^ 299 patients from deCODE (Iceland); subjects from AGRE and Boston Children’s Hospital overlap other studies and were not included here.

^c^ One family (2072) was already reported in Sebat *et al*. [5] (89-3524-100) and Moessner *et al*. [6] (3524), and was not included here.

There have been five studies in ASD that have examined *SHANK3* for mutations, using targeted resequencing (Table 2 and Figure 1). These studies identified five *de novo* deleterious mutations in 1,614 subjects with ASD (0.31%). The combined rate of deletions and mutations in ASD is therefore 0.5%, making haploinsufficiency at the *SHANK3* locus one of the more common monogenic causes of ASD. Studies in intellectual disability and developmental delay confirm this rate of *SHANK3* haploinsufficiency in these disorders as well [19-21].

**Table 2:** ***De novo SHANK3 *mutations identified through large-scale screening of autism spectrum disorder samples**

**Study**	**Subjects**	**Mutations**	**Nucleotide**^**a**^	**Protein**^**b**^	**Exon/intron**
Durand *et al*. [2]	227	1	g.51159940-51159941insG	p.A1227fs	exon 21
Moessner *et al.*[6]	400	1	g.51121844A>G	p.Q321R	exon 8
Gauthier *et al*. [22]	427	1	g.51153476delG	(splice site deletion)	intron 19
Schaaf *et al*. [23]	339	0			
Boccuto *et al*. [24]	221	2	g.51117094C>G	p.P141A	exon 4
			g.51160144delG	p.E1295fs	exon 21
**Total**	**1,614**	**5 (0.31%)**			

^a^ Genomic locations are based on GRCh37 (hg 19). ^b^*SHANK3* reference sequence NM_033517.1 (mRNA) and NP_277052.1 (protein).

**Figure 1:**
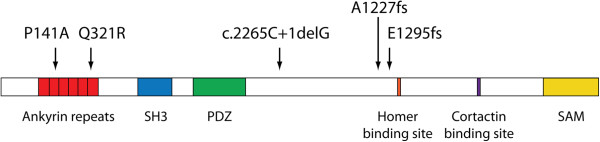
***De novo SHANK3 *mutations identified through large-scale surveys in autism spectrum disorders.** See Table 2 for references.

In conclusion, recent studies of patients with ASD indicate that *SHANK3* haploinsufficiency is found in approximately 0.5% of individuals with ASD. In addition, Soorya and colleagues and prior publications indicate that a very high proportion of individuals with *SHANK3* haploinsufficiency have ASD.

Chromosome microarray analysis is still not routinely carried out for individuals with unexplained developmental delay or ASD, in spite of recommendations from several expert societies. In addition, *SHANK3* is one of the most GC-rich genes in the genome, and targeted resequencing requires considerable optimization to reliably sequence this gene. As a result, few clinical laboratories screen *SHANK3* routinely. Furthermore, whole exome sequencing does a very poor job of adequately covering *SHANK3* because of the GC content. Thus, both clinical and research studies will need to continue to use chromosome microarray analyses and Sanger methods to query this important gene, until better whole-exome or whole-genome sequencing protocols are developed. For all these reasons, Phelan-McDermid syndrome remains undiagnosed in many individuals, denying them and their families any benefits that derive from an etiological diagnosis. As Phelan-McDermid syndrome continues to be studied we will understand more about this disorder, including natural history and therapies that are most beneficial for this group of individuals.

### Abbreviations

ASD: Autism spectrum disorders

### Competing interests

CB and JDB are co-authors of the paper by Soorya and colleagues.
